# Is reactivation of toxoplasmic retinochoroiditis associated to increased annual rainfall?

**DOI:** 10.1051/parasite/2013044

**Published:** 2013-11-14

**Authors:** Marcelo Rudzinski, Alejandro Meyer, Marina Khoury, Cristóbal Couto

**Affiliations:** 1 Uveítis section, Rudzinski oftalmología Oberá Misiones Argentina; 2 APTM clinic 25 de mayo Misiones Argentina; 3 Instituto de Investigaciones Médicas Alfredo Lanari, University of Buenos Aires , Argentina; 4 Uveitis section, Department of Ophthalmology, “Jose de San Martin” Clinical hospital, University of Buenos Aires Argentina

**Keywords:** *Toxoplasma gondii*, retinochoroiditis, rainfall, reactivation, Argentina

## Abstract

**Background:** Reactivation of toxoplasmic retinochoroiditis is the most frequent form of uveitis in Misiones, Argentina. Fluctuations in the number of patients consulting with this type of uveitis were detected during the last decade. Since the province was consecutively exposed to rainy and dry periods over the last years, we decided to explore whether a relationship between reactivation of toxoplasmic retinochoroiditis and rain might be established according to the data registered during the 2004–2010 period. **Results:** The frequency of toxoplasmic reactivation episodes increases when precipitation increases (mostly in second and fourth trimesters of each year). Analysis of the independent variables demonstrates that precipitation is a significant predictor of the frequency of reactivation episodes. Although registered toxoplasmic reactivations were more frequent during the third trimester of the year, the association between the third trimester and the reactivation episodes did not reach statistical significance. **Conclusion:** Prolonged and intense rainfall periods were significantly associated with the reactivation of toxoplasmic retinochoroiditis. Changes promoted by this climatic condition on both the parasite survival in the soil as well as a putative effect on the host immune response due to other comorbidities are discussed.

## Abstract (fr)

**Introduction :** La réactivation de la rétinochoroïdite toxoplasmique est la forme la plus fréquente d’uvéite à Misiones, Argentine. Des fluctuations du nombre de patients consultant avec ce type d’uvéite ont été constatées pendant la dernière décennie. Comme la province a été exposée à des périodes pluvieuses et sèches consécutives pendant les dernières années, nous avons décidé d’explorer si une relation entre la réactivation de rétinochoroïdite toxoplasmique et la pluie pouvait être établie en fonction des données enregistrées au cours de la période 2004–2010. **Résultats :** la fréquence des épisodes de réactivation toxoplasmique croît lorsque les précipitations augmentent (surtout dans les deuxième et quatrième trimestres de chaque année). L’analyse des variables indépendantes démontre que les précipitations sont un facteur prédictif significatif de la fréquence des épisodes de réactivation. Les réactivations toxoplasmiques enregistrées étaient plus fréquentes au cours du troisième trimestre de l’année mais l’association entre le troisième trimestre et les épisodes de réactivation n’a pas atteint une signification statistique. **Conclusion :** les périodes de pluie intense et prolongée étaient significativement associés à la réactivation de la rétinochoroïdite toxoplasmique. Les changements produits par ces conditions climatiques sur la survie du parasite dans le sol ainsi qu’un effet putatif sur la réponse immunitaire de l’hôte dû à d’autres comorbidités sont discutées.

## Introduction

Toxoplasmosis is a disease produced by *Toxoplasma gondii* (Nicolle & Manceaux, 1908) [28] infection that affects almost every warm blooded animal. Humans may acquire the parasite after birth (acquired toxoplasmosis) or from their mother during pregnancy (congenital toxoplasmosis). The eye is frequently affected by the parasite, producing an intraocular inflammation (uveitis). The retina is primarily involved by the invasion of the parasite, while the choroid presents secondary inflammation, a condition referred to as retinochoroiditis. After approximately 60 days, the immune system and/or treatment resolve the retinochoroidal inflammation, leaving a scar from remnants of retinochoroidal tissue, which begins centripetal pigmentation (from the borders toward the center) [19].

At the retinochoroidal scar the slow multiplying bradyzoite form of the parasite can evolve into the fast tissue multiplying form of the parasite, the tachyzoite. When this transformation takes place, from the pigmented border of the retinochoroidal scar, a new neighboring retinal area becomes inflamed, initiating a period of parasite reactivation.

**Figure 1.**
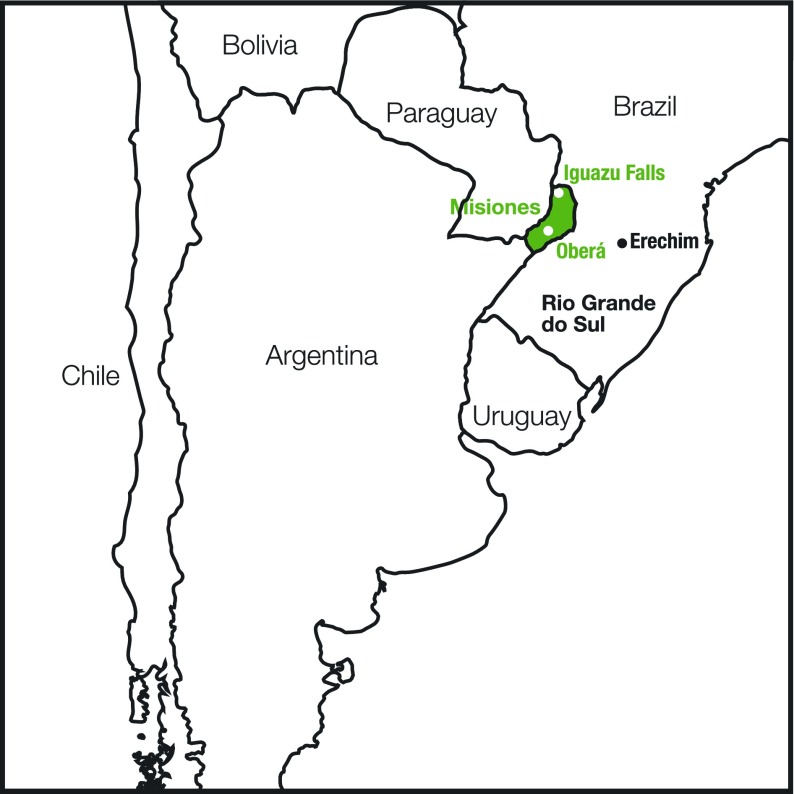
Map of South America. The province of Misiones is located in the extreme northeast of Argentina, between Paraguay and Brazil. The city of Oberá is at the center of the province. Misiones shares its eastern border with the Brazilian states of Rio Grande do Sul and Santa Catarina. The former state is known for its high prevalence of ocular toxoplasmosis.

Toxoplasmosis is a prevalent disease in Argentina [5, 8]. Misiones, a province located in the extreme Northeastern region of Argentina (Figure 1), shares its Eastern borders with the neighbor Southern Brazilian states of Rio Grande do Sul, Santa Catarina, and Parana, a highly endemic toxoplasmosis region [40, 41]. The latter states also share similar geological and climatic characteristics with Misiones. In this regard, Misiones soil belongs to the Brazilian massif and its surface is covered by the Atlantic rainforest. Moreover, according to the National Weather Institute (Servicio Meteorológico Nacional, SMN) the climate of Misiones is subtropical, without any dry season. Annual precipitation ranges from 1,000 to 2,000 mm in the center part of the province. Rainfall shows two well-defined peaks, one occurring in April and a second and more important one during springtime (September 21st–December 21st period). The city of Oberá (250 km away from Iguazu Falls) is placed in the center region of Misiones Province; it exhibits a mean annual temperature of 20.8 C. Rain and humidity air levels are not completely homogeneous in Misiones. They reach maximum values in the northeast part of the province and their lowest values in the southeast. This difference is explained due to the arrival of moisture from the Atlantic Ocean to the eastern and central hills of the province.

During the Southern oscillation called “El Niño” phenomena, when the Pacific Ocean waters in front of the Peruvian coast increase its temperatures (starting at the end of the austral winter), there is an increased rainfall during spring time in Misiones. As expected, during “La Niña” phenomena, a period of dryness also affects this province.

Prevalence of serum antibodies against *T. gondii* is around 80% among inhabitants living in the central-east region of the province. More importantly, recent data indicates that 20% of examined patients at the ophthalmic consulting clinic in the above-mentioned region, exhibits toxoplasmic retinochoroidal scars [33]. Similar results have been reported in the nearby city of Erechim, Rio Grande do Sul, few miles away from the Argentinean border [15]. In Oberá as in the neighbor Brazilian state, more than 90% of the patients with a diagnosis of posterior uveitis have ocular toxoplasmosis. Most of those cases are reactivation of toxoplasmic retinochoroidal scars. Primary infection of the retinochoroidal tissue by the parasite is usually asymptomatic. Symptomatic cases attending the clinic with primary ocular toxoplasmosis represent less than 10% of the ocular toxoplasmosis cases.

Taking into account that we have annually observed variations in the number of patients attending the ophthalmic clinic with reactivation of toxoplasmic retinochoroiditis and that Misiones has been consecutively exposed to “La Niña” and “El Niño” periods, we decided to explore whether a relationship between total annual rainfall and reactivation of toxoplasmic retinochoroiditis could be recorded.

## Material and methods

The study was conducted at Rudzinski Oftalmologia in Oberá, Misiones, a second referral clinic. At this clinic, there is one retinal specialist, one uveitis specialist, and one glaucoma specialist. Patients with uveitis are examined by a single ophthalmologist specialized in uveitis (MR). Uveitis patients usually undergo complete ophthalmological examination including visual acuity, anterior biomicroscopy, tonometry, and indirect ophthalmoscopy. Patients showing atypical clinical manifestations, such as extensive necrotizing retinochoroiditis, bilateral retinochoroiditis, and/or extensive vasculitis, were investigated for HIV infection as well as the possibility of a concomitant immunosuppressive treatment.

Clinical history of patients attending the ophthalmological clinic during the period January 2004–December 2010 and fulfilling the case definition of reactivation of toxoplasmic retinochoroiditis episode was reviewed. Patients with a pigmented retinal scar surrounded by a white inflamed retinal area (wide through retinal lesion, with slightly elevated, edematous retina), with vitreitis (vitreous humor inflammation), with or without vasculitis (inflammation of the vessels), with or without papillitis (inflammation of the optic nerve), and with or without signs of anterior chamber inflammation that responded well to pyrimethamine (25 mg/d) + sulfadiazine (1.5 g/d) and meprednisone (20–40 mg/d) were considered as toxoplasmic reactivation episode. Patients presenting negative results for anti-*T. gondii* IgG were excluded. Data from 2007 was not included, since the uveitis specialist had gone abroad as a postdoctoral fellow.

No campaigns encouraging the examination of patients with ocular toxoplasmosis were conducted during the period of study at the clinic.

Detailed daily precipitation from year 2004 to 2010 was obtained from the Weather Information Center (CIM, Centro de Información Metereológica) from the SMN. The original rainfall data was measured at the SMN weather base in Oberá, province of Misiones.

Results are presented as mean ± standard deviation or median and range for numerical variables and absolute frequency for categorical variables.

To analyze the association between the number of toxoplasmic reactivations as dependent variable and precipitation (per trimester) as independent variable, a Poisson regression model was used. Due to the fact that 2007 data was not available, a time series model was not feasible. To adjust for seasonality, the trimester of the year was included as a second independent variable in the Poisson model. Data are presented as odds ratio (OR) and 95% confidence interval (CI). Analysis was performed using IBM SPSS Statistics [20].

## Results

Eighty-one patients were included in the analysis. Forty-two of the examined charts belonged to female patients. Mean age ± *SD* of the whole population was 35.3 ± 15.7 yr-old, while the median age was 31.0 yr-old. Five patients had two reactivation episodes. Eighty-six reactivation episodes of toxoplasmic retinochoroiditis were identified.

Median total annual rain was 1580.83 mm (range = 1,290–1,881) during the period of study.

**Figure 2.**
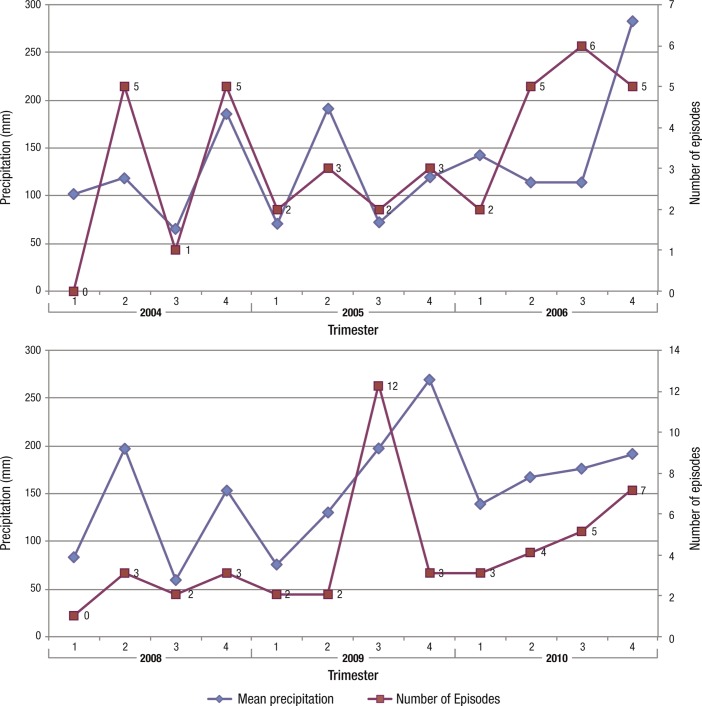
Graphic showing quarterly precipitation and toxoplasmic reactivation of retinochoroiditis detected episodes in Oberá, during the years 2004–2006 (superior) and 2008–2010 (inferior). The frequency of reactivation of toxoplasmic retinochoroiditis increases when precipitation increases. Highest frequency episodes took place during “El Nino” phenomena (2006 and 2009).

Reactivation episodes and rain precipitations did not occur within the same frequency all year around (Figure 2). The frequency of toxoplasmic reactivation episodes increases when precipitation increases (mostly in second and fourth trimesters of each year). Higher precipitation marks and the highest number of reactivation cases are observed during “El Niño” phenomena years (2006 and 2009). Analysis of the independent variables demonstrates that precipitation is a significant predictor of the frequency of reactivation episodes. For every mm of precipitation there is a 2‰ increase in episodes (see Table 1).

**Table 1.** Poisson regression model indicates precipitation as a significant predictor of reactivation of toxoplasmic retinochoroiditis (*p* = 0.019). Although toxoplasmic retinochoroiditis reactivation episodes happen more frequently during the third trimester of the year, there was not statistically significant association between that trimester of the year and the reactivation episodes.

Parameter	*B* Coefficient	Odds ratio	95% CI		*P* value
Precipitation (mm)	0.002	1.002	1.000	1.003	0.019
4th trimester (reference)	0	1			
3rd trimester	0.549	1.732	0.804	3.729	0.161
2nd trimester	0.12	1.128	0.672	1.893	0.650
1st trimester	−0.202	0.817	0.350	1.908	0.641
Intercept	0.342				0.509

Although the result was statistically not significant, it should be distinguished that the third trimester of the year had an odds ratio of 1.73 times that of the 4th trimester (reference group).

## Discussion

Reactivation of toxoplasmic retinochoroiditis is the most frequent clinical form of presentation of the ocular disease produced by this parasite. Reactivation of toxoplasmic disease is the consequence of several factors inherent to the host (immune status and genetic background) and to the parasite (genetic strain).

The possibility that host immunosuppression could result in reactivation of toxoplasmic retinochoroiditis was established since the 1980s [2, 3, 18, 25, 30, 37]. Regarding the parasite, an association between genotypic strains of the parasite and severity of clinical disease was analyzed over the last decade. In Western Europe, the severity of the ocular disease is low and has been associated to a predominance of genotype type II on intraocular fluids of patients with retinochoroiditis [12]. In South America, retinochoroiditis reactivation episodes are more frequent and severe than in European and North American patients possibly due to the presence of more genetically divergent (nonclonal) parasite strains in ocular samples [13, 16, 21]. The abundance of genetically divergent strains of the parasite in this region could also explain the possibility of reinfection. It has been demonstrated that immune response against one strain may not be completely protective against a different strain [10].

In the present study, the frequency of toxoplasmic reactivation episodes per trimester of the year in patients from the subtropical province of Misiones, Argentina is described. The result suggests an association between precipitation as rain and reactivation of toxoplasmic retinochoroiditis. Rainfall affects the *T. gondii* cycle in nature. Oocyst survival is higher in humid warm soil than under dry and high temperature conditions [7, 22]. Moreover, humidity may also increase its potential infectivity, since under humid and warm temperature conditions the oocysts may sporulate and become infective in one day [6]. Hence, not surprisingly, warm countries with a high annual rainfall are also the places where seropositivity for animals and humans is the highest [34, 35, 45]. In France, rain increases the risk of seroconversion for *T. gondii* in cats [1]. As expected, it also affects human infections. A recent study from Colombia showed a strong correlation between the highest mean annual rainfall and an increase in the incidence for markers of congenital toxoplasmosis in pregnant women [17]. Similar observations were made in the Rhone-Alpes region of France, were congenital toxoplasmosis was shown to occur more frequently at the end of the summer, when the climate gets warmer and humid [27].

### How could an increased annual rainfall affect the frequency of reactivation of toxoplasmic retinochoroiditis?

Humidity and mild temperatures promote respiratory viral infections, which are frequent in Misiones during winter and spring time, coincidently with the period when reactivation of toxoplasmic retinochoroiditis is more frequent. Some respiratory viruses are known to induce transient immunosuppressive states, such as a decrease of white blood cells. Although our patients did not clinically reflect symptoms of any moderate or severe respiratory infection at the moment of consultation, such as frequent cough and fever, we cannot rule out the possibility that some of the patients may had been experiencing an asymptomatic respiratory viral infection that could facilitate the reactivation of toxoplasmic retinochoroiditis. Argentina, including Misiones, experienced an epidemic of influenza A H1N1 during winter and spring time of 2009 [9, 23], one of the years with highest registered cases of reactivation of toxoplasmic retinochoroiditis (SMN).

On the other hand, rainfall contributes to the spread of the potential infective oocysts in the surrounding areas into faster flowing water streams. It is because of this that during the rainfall period, people from affected regions could be exposed to more frequent parasite ingestion episodes. Based on the relevance of the immune system status on reactivation of toxoplasmic retinochoroiditis it is tempting to hypothesize that frequent parasite reingestion could lead to chronic changes in the immune system that could facilitate reactivation of retinochoroiditis. There is evidence that chronic oral exposure to an antigen can induce a state of systemic immune tolerance for a specific antigen on some patients [4, 39]. Different oral antigens are currently in use for the prevention of uveitis relapse in autoimmune diseases such as the Behçet disease [36]. Systemic tolerance to a specific orally administered antigen has been shown to be mediated through CD4^+^ CD25^+^ T regulatory cells [38, 42]. The regulatory activity on Th1, Th2, and CD8^+^ cells is mediated through the local secretion of TGF-ß ad IL-10 [44]. In the eye, infiltrates of CD4^+^ CD25^+^ T cells are specifically found in the retina of patients with recently acquired toxoplasmosis indicating that recently orally ingested parasite leads to the presence of these immune-regulatory cells in their infected retinas [26]. Their presence was explained as a mechanism to modulate the retinal damage, through a regulatory effect on the Th-1 response during the retinochoroiditis [14]. However a recent paper described the acquisition of transcription factors by CD4^+^ CD25^+^ T regulatory cells along the pathway to the homing organ affecting its future regulatory activity [29, 43]. On the view of such new results it is tempting to speculate that frequent *Toxoplasma gondii* ingestion could lead to a change in the activity of CD4^+^ CD25^+^ regulatory cells in the retina and consequently interfere with the equilibrium between the quiescent bradyzoites and the immune state of the eye leading to reactivation of the retinochoroiditis.

Another possible explanation on how rain can facilitate reactivation of toxoplasmic retinochoroiditis is related to the lack of sunlight during prolonged rainy periods. The active form of Vitamin D3, 1, 25 (OH) two vitamin D3 is formed after its precursor is activated on the patient’s skin exposed to UV spectrum of the sunlight. The active form of vitamin D3 was shown to reduce the in vivo and in vitro intracellular growth of *T. gondii* [32]. More recently, active vitamin D3 was also shown to induce the secretion of IFN gamma [11], a key mediator in *T. gondii* infection control. Although South American patients have enough sunlight exposure most of the year, they lack it during long periods of rain and also do not have enough levels of its precursor, inactive vitamin D3, through their regular diets [31]. Cathelicidin, a peptide pathway stimulated by active vitamin D3, is a well-known mechanism of clearance of intracellular microorganisms in neutrophils and macrophages [24]. As a result of prolonged rainy periods, persistence of a high number of intracellular parasites due to inactive cathelicidin pathway could possibly lead to more frequent reactivations of toxoplasmic retinochoroiditis.

Our results, adjusted for trimester of the year, show that reactivation of toxoplasmic retinochoroiditis occurs more frequently during rainy periods. Although the result did not reach statistical significance, the third trimester presented larger number of reactivations and consequently seasonal variations could not be ruled out.

The results we reported stimulate us to pursue specific explanations. Limitation of the study includes the lack of data in 2007, and need for adjustment for other possibly related variables. More studies are needed to explain the effect of rain on reactivation of toxoplasmic retinochoroiditis. The validation of these findings would allow the implementation of strategies to assure treatment access during rainy periods as well as prevention campaigns.

## References

[1] Afonso E, Thulliez P, Gilot-Fromont E. 2010 Local meteorological conditions, dynamics of seroconversion to *Toxoplasma gondii* in cats (*Felis catus*) and oocyst burden in a rural environment. Epidemiology and Infection, 138, 1105–1113 19961642 10.1017/S0950268809991270

[2] Basu S, Das T, Biswas G. 2010 Bilateral toxoplasma retinochoroiditis in a patient with chronic myeloid leukemia treated with imatinib mesylate. Ocular Immunology and Inflammation, 18, 64–65 20128654 10.3109/09273940903315792

[3] Bottós J, Miller RH, Belfort RN, Macedo AC, UNIFESP Toxoplasmosis Group, Belfort R Jr, Grigg ME. 2009 Bilateral retinochoroiditis caused by an atypical strain of *Toxoplasma gondii*. British Journal of Ophthalmology, 93, 1546–1550 19666926 10.1136/bjo.2009.162412 PMC4029948

[4] Chen Y, Inobe J, Marks R, Gonnella P, Kuchroo VK, Weiner HL. 1995 Peripheral deletion of antigen-reactive T cells in oral tolerance. Nature, 376, 177–180 7603570 10.1038/376177a0

[5] Del Vado G. 1997 Congenital toxoplasmosis in South American children. Archivos Argentinos de Pediatria, 95, 14–20

[6] Dubey JP, Frenkel JK. 1972 Cyst induced toxoplasmosis in cats. Journal of Protozoology, 19, 155–177 5008846 10.1111/j.1550-7408.1972.tb03431.x

[7] Dubey JP. 1998 *Toxoplasma gondii* oocyst survival under defined temperatures. Journal of Parasitology, 84, 862–865 9714227

[8] Durlach R, Kaufer F, Carral L, Freuler C, Ceriotto M, Rodriguez M, Freilij H, Altech J, Vazquez L, Corazza R, Maria Dalla Fontana, Arienti H, Sturba E, Gonzalez Ayala S, Cecchini E, Salomon C, Nadal M, Gutierrez N, Guarnera E. 2008 Consenso Argentino de Toxoplasmosis Congénita. Medicina (Buenos Aires), 68, 75–87 18416325

[9] Echavarría M, Querci M, Marcone D, Videla C, Martinez A, Bonvehi P, Carballal G. 2010 Pandemic (H1N1) 2009 cases, Buenos Aires, Argentina. Emerging Infectious Diseases, 16, 311–313 20113568 10.3201/eid1602.091114 PMC2958018

[10] Elbez-Rubinstein A, Ajzenberg D, Dardé ML, Cohen R, Dumètre A, Yera H, Gondon E, Janaud JC, Thulliez P. 2009 Congenital toxoplasmosis and reinfection during pregnancy: case report, strain characterization, experimental model of reinfection, and review. Journal of Infectious Diseases, 199, 280–285 19032062 10.1086/595793

[11] Fabri M, Stenger S, Shin DM, Yuk JM, Liu PT, Realegeno S, Lee HM, Krutzik SR, Schenk M, Sieling PA, Teles R, Montoya D, Iyer SS, Bruns H, Lewinsohn DM, Hollis BW, Hewison M, Adams JS, Steinmeyer A, Zügel U, Cheng G, Jo EK, Bloom BR, Modlin RL. 2011 Vitamin D is required for IFN-gamma-mediated antimicrobial activity of human macrophages. Science Translational Medicine, 3, 104ra102 10.1126/scitranslmed.3003045 PMC3269210 21998409

[12] Fekkar A, Fekkar A, Ajzenberg D, Bodaghi B, Touafek F, Le Hoang P, Delmas J, Robert PY, Dardé ML, Mazier D, Paris L. 2011 Direct genotyping of *Toxoplasma gondii* in ocular fluid samples from 20 patients with oculartoxoplasmosis: predominance of type II in France. Journal of Clinical Microbiology, 49, 1513–1517 21248092 10.1128/JCM.02196-10 PMC3122785

[13] Ferreira IM, Vidal JE, de Mattos Cde C, de Mattos LC, Qu D, Su C, Pereira-Chioccola VL. 2011 *Toxoplasma gondii* isolates: multilocus RFLP-PCR genotyping from human patients in Sao Paulo State, Brazil identified distinct genotypes. Experimental Parasitology, 129, 190–195 21741380 10.1016/j.exppara.2011.06.002

[14] Garweg JG, Candolfi E. 2009 Immunopathology in ocular toxoplasmosis: facts and clues. Memorias do Instituto Oswaldo Cruz, 104, 211–220 19430646 10.1590/s0074-02762009000200014

[15] Glasner PD, Silveira C, Kruszon-Moran D, Martins MC, Burnier Júnior M, Silveira S, Camargo ME, Nussenblatt RB, Kaslow RA, Belfort Júnior R. 1992 An unusually high prevalence of ocular toxoplasmosis in southern Brazil. American Journal of Ophthalmology, 114, 136–144 1642287 10.1016/s0002-9394(14)73976-5

[16] Gilbert RE, Freeman K, Lago EG, Bahia-Oliveira LM, Tan HK, Wallon M, Buffolano W, Stanford MR, Petersen E, European Multicentre Study on Congenital Toxoplasmosis (EMSCOT) 2008 Ocular sequelae of congenital toxoplasmosis in Brazil compared with Europe. PLoS Neglected Tropical Diseases, 2, e277 18698419 10.1371/journal.pntd.0000277 PMC2493041

[17] Gomez-Marin JE. 2011 First Colombian multicentric newborn screening for congenital toxoplasmosis. PLoS Neglected Tropical Diseases, 5, e1195 21655304 10.1371/journal.pntd.0001195 PMC3104965

[18] Holland GN, Engstrom RE Jr, Glasgow BJ, Berger BB, Daniels SA, Sidikaro Y, Harmon JA, Fischer DH, Boyer DS, Rao NA, Eagle RC Jr, Kreiger AE, Foos RY. 1988 Ocular toxoplasmosis in patients with acquired immunodeficiency syndrome. American Journal of Ophthalmology, 106, 653–667 3195645 10.1016/0002-9394(88)90697-6

[19] Holland GN, O’Connor GR, Belfort R Jr. 1995 Toxoplasmosis ocular infection and immunity, Chapter 85. Pepose Jay S, Holland Gary N, Whilhelmus Kirk R (Eds.), 1183–1223,

[20] IBM Corp. Released 2010 IBM SPSS Statistics for Windows, Version 19.0, Armonk, NY: IBM Corp

[21] Khan A, Jordan C, Muccioli C, Vallochi AL, Rizzo LV, Belfort R Jr, Vitor RW, Silveira C, Sibley LD. 2006 Genetic divergence of *Toxoplasma gondii* strains associated with ocular toxoplasmosis, Brazil. Emerging Infectious Diseases, 12, 942–949 16707050 10.3201/eid1206.060025 PMC3373049

[22] Lélu M, Villena I, Dardé ML, Aubert D, Geers R, Dupuis E, Marnef F, Poulle ML, Gotteland C, Dumètre A, Gilot-Fromont E. 2012 Quantitative estimation of the viability of *Toxoplasma gondii* oocysts in soil. Applied Environmental Microbiology, 78, 5127–5132 22582074 10.1128/AEM.00246-12 PMC3416395

[23] Libster R, Bugna J, Coviello S, Hijano DR, Dunaiewsky M, Reynoso N, Cavalieri ML, Guglielmo MC, Areso MS, Gilligan T, Santucho F, Cabral G, Gregorio GL, Moreno R, Lutz MI, Panigasi AL, Saligari L, Caballero MT, Egües Almeida RM, Gutierrez Meyer ME, Neder MD, Davenport MC, Del Valle MP, Santidrian VS, Mosca G, Garcia Domínguez M, Alvarez L, Landa P, Pota A, Boloñati N, Dalamon R, Sanchez Mercol VI, Espinoza M, Peuchot JC, Karolinski A, Bruno M, Borsa A, Ferrero F, Bonina A, Ramonet M, Albano LC, Luedicke N, Alterman E, Savy V, Baumeister E, Chappell JD, Edwards KM, Melendi GA, Polack FP. 2010 Pedriatic hospitalizations associated with 2009 pandemic influenza A (H1N1) in Argentina. New England Journal of Medicine, 362, 45–55 20032320 10.1056/NEJMoa0907673

[24] Liu PT, Stenger S, Li H, Wenzel L, Tan BH, Krutzik SR, Ochoa MT, Schauber J, Wu K, Meinken C, Kamen DL, Wagner M, Bals R, Steinmeyer A, Zügel U, Gallo RL, Eisenberg D, Hewison M, Hollis BW, Adams JS, Bloom BR, Modlin RL. 2006 Toll-like receptor triggering of a vitamin D-mediated human antimicrobial response. Science, 311, 1770–1773 16497887 10.1126/science.1123933

[25] Mele A, Paterson PJ, Prentice HG, Leoni P, Kibbler CC. 2002 Toxoplasmosis in bone marrow transplantation: a report of two cases and systematic review of the literature. Bone Marrow Transplantation, 29, 691–698 12180115 10.1038/sj.bmt.1703425

[26] Miyamoto C, Rubens Belfort M, Di Cesare S, Rubens Belfort J, Burnier MN Jr. 2010 Use of CD25 as an immunohistochemical marker for acquired toxoplasmosis. Arquivos Brasileiros de Oftalmologia, 73, 443–446 21225130 10.1590/s0004-27492010000500011

[27] Morin L, Lobry Jr, Peyron F, Wallon M. 2012 Seasonal variations in acute toxoplasmosis in pregnant women in the Rhone-Alpes region (France). Clinical Microbiology and Infection, 10, 401–403 10.1111/j.1469-0691.2012.03898.x 22616769

[28] Nicolle C, Manceaux L. 1909 Sur un protozoaire nouveau du gondi. Comptes Rendus des Séances de l’Académie des Sciences, 148, 369–372

[29] Oldenhove G, Bouladoux N, Wohlfert EA, Hall JA, Chou D, Dos Santos L, O’Brien S, Blank R, Lamb E, Natarajan S, Kastenmayer R, Hunter C, Grigg ME, Belkaid Y. 2009 Decrease of Foxp3^+^ Treg cell number and acquisition of effector cell phenotype during lethal infection. Immunity, 31, 772–786 19896394 10.1016/j.immuni.2009.10.001 PMC2814877

[30] Park KL, Smith RE, Rao NA. 1995 Ocular manifestations of AIDS. Current Opinion in Ophthalmology, 6, 82–87 10160424 10.1097/00055735-199512000-00014

[31] Portela ML, Mónico A, Barahona A, Dupraz H, Sol Gonzales-Chaves MM, Zeni S. 2010 Comparative 25-OH-vitamin D level in institutionalized women older than 65 years from two cities in Spain and Argentina having a similar solar radiation index. Nutrition, 26, 283–289 19819110 10.1016/j.nut.2009.04.022

[32] Rajapakse R, Uring-Lambert B, Andarawewa KL, Rajapakse RP, Abou-Bacar A, Marcellin L, Candolfi E. 2007 1,25(OH)2D3 inhibits in vitro and in vivo intracellular growth of apicomplexan parasite *Toxoplasma gondii*. Journal of Steroid Biochemistry, 103, 811–814 10.1016/j.jsbmb.2006.12.058 17270431

[33] Rudzinski M, Meyer A. 2011 Prevalence and risk factors associated with ocular toxoplasmosis in patients from the center east region of the province of Misiones, Argentina. Oftalmología Clínica y Experimental, 4, 159–162

[34] Singh S, Pandit AJ. 2004 Incidence and prevalence of toxoplasmosis in Indian pregnant women: a prospective study. American Journal of Reproductive Immunology, 52, 276–283 15494049 10.1111/j.1600-0897.2004.00222.x

[35] Sroka S, Bartelheimer N, Winter A, Heukelbach J, Ariza L, Ribeiro H, Oliveira FA, Queiroz AJ, Alencar C Jr, Liesenfeld O. 2010 Prevalence and risk factors of toxoplasmosis among pregnant women in Fortaleza, North-eastern Brazil. American Journal of Tropical Medicine and Hygiene, 83, 528–533 20810816 10.4269/ajtmh.2010.10-0082 PMC2929047

[36] Stanford M, Whittal T, Bergmeier LA, Lindbland M, Lundin S, Shinnick T, Mizushima Y, Holmgren J, Lehner T. 2004 Oral tolerization with peptide 336–51 link to cholera toxin B in preventing relapses of uveitis in Behcet’s disease. Clinical and Experimental Immunology, 137, 201–208 15196263 10.1111/j.1365-2249.2004.02520.x PMC1809095

[37] Theaudin M, Bodaghi B, Cassoux N, Romand S, Le Mer Y, Lemaitre C, Fardeau C, Thulliez P, LeHoang P. 2003 Extensive toxoplasmic retinochoroiditis. Diagnostic and therapeutic management. Journal Français d’Ophtalmologie, 26, 921–927 14631276

[38] Thorstenson KM, Khoruts A. 2001 Generation of anergic and potentially immunoregulatory CD25+CD4 T cells in vivo after induction of peripheral tolerance with intravenous or oral antigen. Journal of Immunology, 167, 188–195 10.4049/jimmunol.167.1.188 11418648

[39] Van Wilsem EJ, Van Hoogstraten IM, Brevé J, Scheper RJ, Kraal G. 1994 Dendritic cells of the oral mucosa and the induction of oral tolerance. A local affair. Immunology, 83, 128–132 7821957 PMC1415022

[40] Varella IS, Canti IC, Santos BR, Coppini AZ, Argondizzo LC, Tonin C, Wagner MB. 2009 Prevalence of acute *Toxoplasma gondii* infection among 41112 pregnant women and the mother to child transmission rate in a public hospital in south Brazil. Memorias do Instituto Oswaldo Cruz, 104, 383–388 19430669 10.1590/s0074-02762009000200037

[41] Vaz RS, Thomaz-Soccol V, Sumikawa E, Guimaraes AT. 2010 Serologic prevalence of *Toxoplasma gondii* antibodies in pregnant women of southern Brazil. Parasitology Research, 106, 661–665 20084396 10.1007/s00436-009-1716-2

[42] Weiner HL. 2001 Induction and mechanism of action of transforming growth factor-beta-secreting Th3 regulatory cells. Immunological Reviews, 182, 207–214 11722636 10.1034/j.1600-065x.2001.1820117.x

[43] Wohlfert E, Belkaid Y. 2010 Plasticity of T reg at infected sites. Mucosal Immunology, 3, 213–215 20237465 10.1038/mi.2010.11 PMC3415210

[44] Zhang X, Izikson L, Liu L, Weiner HL. 2001 Activation of CD25(+)CD4(+) regulatory T cells by oral antigen administration. Journal of Immunology, 167, 4245–4253 10.4049/jimmunol.167.8.4245 11591746

[45] Zemene E, Yewhalaw D, Abera S, Belay T, Samuel A, Zeynudin A. 2012 Seroprevalence of *Toxoplasma gondii* and associated risk factors among pregnant women in Jimma town, Southwestern Ethiopia. BMC Infectious Diseases, 12, 337 DOI:10.1186/1471-2334-12-337 23216887 10.1186/1471-2334-12-337 PMC3519766

